# Differential xylem phytohormone export from dry and wet roots during partial rootzone drying is independent of shoot‐to‐root transport in soybean

**DOI:** 10.1111/ppl.70252

**Published:** 2025-04-29

**Authors:** Jaime Puértolas, Pedro Castro‐Valdecantos, Alfonso Albacete, Ian C. Dodd

**Affiliations:** ^1^ Lancaster Environment Centre Lancaster University Lancaster United Kingdom; ^2^ Departamento de Producción Vegetal y Agrotecnología Instituto Murciano de Investigación y Desarrollo Agrario y Alimentario (IMIDA) La Alberca Murcia Spain; ^3^ Present address: Department of Botany and Plant Ecology and Physiology University of La Laguna La Laguna Canary Islands Spain; ^4^ Present address: Universidad de Sevilla, Área de Ingeniería Agroforestal, Dpto. de Ingeniería Aeroespacial y Mecánica de Fluidos, Escuela Técnica Superior de Ingeniería Agronómica, University of Seville Seville Spain

## Abstract

Different phytohormones can act as root‐to‐shoot signalling molecules in response to soil drying. Recent findings suggest that root ABA levels are predominantly leaf‐sourced and not locally synthesized, thus, ABA exported from the roots in the xylem is mostly recycled from the shoot. To explain the differential root hormone accumulation observed under partial rootzone drying (PRD) that imposes distinct dry and wet parts of the root zone, we grafted “two‐root, one‐shoot” soybean plants to independently assess xylem export of different phytohormones from either part of the root zone. Grafts were subjected to a combination of girdling (either part, all, or none of the rootzone) and irrigation (homogenously well‐watered (WW) and PRD). PRD did not increase foliar ABA but decreased stomatal conductance, attributed to decreased leaf water potential and/or increased xylem sap ABA, JA, or ACC concentrations. In contrast, the foliar ABA increments that accompanied girdling‐induced stomatal closure were proportional to the root fraction to which phloem transport was interrupted. Irrespective of girdling, root ABA accumulation (and xylem ABA export from) was highest in the dry PRD rootzone, xylem jasmonic acid (JA) in the wet PRD rootzone, and xylem ACC in both rootzones of PRD plants. Thus, soil drying of the dry root zone and transient overwatering of the wet root zone enhanced ACC export in PRD plants. We conclude that root water status during PRD enhances root ABA, JA and ACC synthesis and xylem export, independent of shoot‐to‐root transport.

## 1 INTRODUCTION

Since roots are in close contact with the soil, understanding how they sense soil water deficit and transmit that information to the shoot to regulate shoot physiology has attracted much research interest (Shabala et al., 2015, Li et al., 2021). Especially the role of long‐distance signalling mediated by root‐sourced phytohormones has been intensively studied (Huntenburg et al. 2022). Early studies on root‐to‐shoot signalling focused on abscisic acid (ABA), as it plays a central role in plant physiological responses to abiotic stress (Roychoudhury et al. 2013; Sakata et al. 2014; Todaka et al. 2019). It accumulates in roots in response to decreasing root water potential even when shoot water status does not change (Zhang et al. 1987; Khalil and Grace 1992; Liu et al. 2005) and is transported to the shoot in the xylem sap (Zhang and Davies 1990; Puértolas et al. 2013). Collectively, these observations suggested that ABA might act as a putative root‐to‐shoot long‐distance signal of soil drying.

Other phytohormones can also act as long‐distance root‐to‐shoot signals of soil drying (Lacombe and Achard 2016). Soil drying enhanced root and xylem sap concentration of the ethylene precursor 1‐aminocyclopropane‐1‐carboxylic acid or ACC (Tudela and Primo‐Millo 1992), jasmonic acid (JA) and other jasmonates (de Ollas et al. 2018), while decreasing root cytokinin export (Bano et al. 1994; Alvarez et al. 2008). While ABA has been the primary target for studies on long‐distance hormonal signalling of drought, other hormones can interact with ABA to modulate stomatal responses (Dodd 2003; Tanaka et al. 2005; Hossain et al. 2011; Arnaud et al. 2017).

Soil moisture is usually distributed unevenly within the root zone due to evaporation from upper soil layers, differences in root density and root water uptake, and sometimes heterogeneous application of irrigation water. Even though some water can be redistributed from roots in wet soil to those in dry soil following root water potential gradients (Stoll et al. 2000), this heterogeneity creates differences in root water potential across the root zone, which determines local variation in phytohormone accumulation (Puértolas et al. 2015). The differential effect of heterogenous soil drying on phytohormone accumulation and xylem export to the shoots is well documented for ABA. Soil moisture gradients that developed vertically in soil columns (Zhang and Davies 1989) and horizontally in split‐root experiments following partial rootzone drying (PRD) that watered only part of the rootzone (Khalil and Grace 1992) caused heterogeneity in root ABA accumulation. However, root‐to‐shoot ABA export also depends on water flows from different parts of the root zone. Prolonged drying of part of the root system decreased sap flow and thus ABA export from those roots (Dodd et al. 2008a), since increased ABA concentration driven by phloem water recirculation into the xylem (Tanner and Beevers 2001) cannot compensate for roots in drying soil contributing proportionally less to overall plant transpiration flow. This explains a model of ABA export to shoots under PRD that incorporated xylem ABA concentration and proportional sap flow from each part of the root system (Dodd et al. 2008a). ABA export from the entire root system was maximal with intermediate soil moisture surrounding the dry roots, allowing root accumulation but also substantial sap fluxes from those roots to transport ABA. Whether this model can be applied to other phytohormones has not been assessed.

Soil moisture heterogeneity also causes differential root accumulation of other hormones and their precursors. Root ACC levels increased in the dry side of tomato plants exposed to PRD, with alternation of the wet and dry sides enhancing root‐to‐shoot ACC signalling and foliar ethylene evolution (Pérez‐Pérez et al. 2020). Concentrations of active cytokinins zeatin and zeatin‐riboside decreased in drying roots of split‐root grapevine (Stoll et al. 2000), and although PRD did not affect xylem zeatin concentrations, decreased root zeatin export partially explained decreased foliar accumulation (Kudoyarova et al. 2007). Partial rootzone drying also increased auxin and JA concentrations in dried roots in olive seedlings (*Olea europaea*), with root‐to‐shoot hormone transport likely causing higher foliar concentrations (Abboud et al. 2021). Thus, differential soil moisture can alter xylem phytohormone export from different parts of the root system, but most studies have focused on a single hormone. Assessing the relative sensitivity of each to soil drying has received limited attention.

Since these phytohormones can also be transported in phloem sap (Weiler and Ziegler 1981), root phytohormone accumulation could depend on transport from the shoot in the phloem sap. ABA concentrations in phloem sap are much higher than in xylem sap (Peuke 2016), thus ABA was proposed to act as a phloem‐borne signal that could increase ABA accumulation in sink tissues (e.g. roots) that were not experiencing water deficits (Zhong et al. 1996; Jeschke et al. 1997). However, reciprocal grafting of wild‐type and ABA‐deficient mutants suggests that root ABA status is mostly shoot‐sourced (McAdam et al. 2016; Li et al. 2018) and that root tissues have very low capacity to synthesise ABA. Nevertheless, earlier works with water‐stressed detached roots (Cornish and Zeevaart 1985; Lachno and Baker 1986; Simonneau et al. 1998; Borel et al. 2001) or girdled plants subjected to soil drying (Cornish and Zeevaart 1985) demonstrated significant autonomous root ABA synthesis. Similarly, in cotton (*Gossypium hirsutum*), both leaf‐ and root‐sourced jasmonic acid contributed to root JA accumulation in osmotically stressed roots comprising half the root zone, but JA increments in the non‐stressed roots were thought to be leaf‐derived as they did not occur in girdled plants (Luo et al. 2019). Therefore, shoot and root hormone synthesis and bidirectional transport between them explain hormone accumulation in different organs of the plant. While recirculation of ABA between phloem and xylem in the roots has been modelled in response to osmotic (salt) stress (Wolf et al. 1990), to our knowledge, shoot‐to‐root transport of other putative hormone root‐to‐shoot signals has not been considered, especially under heterogeneous soil drying.

Differential accumulation of ABA in dry and wet parts of the rootzone observed under PRD agrees with the variation in root water potential measured in each (Puértolas et al. 2015), seemingly confirming the local origin of root ABA. Nevertheless, decreased water uptake from roots in drying soil may limit the xylem export of ABA to the shoots (Dodd et al. 2008b), allowing roots in drying soil to accumulate shoot‐sourced ABA, but this has not been experimentally tested. Likewise, such processes may explain the differential accumulation of other hormones between dry and wet parts of the rootzone during PRD.

**FIGURE 1:**
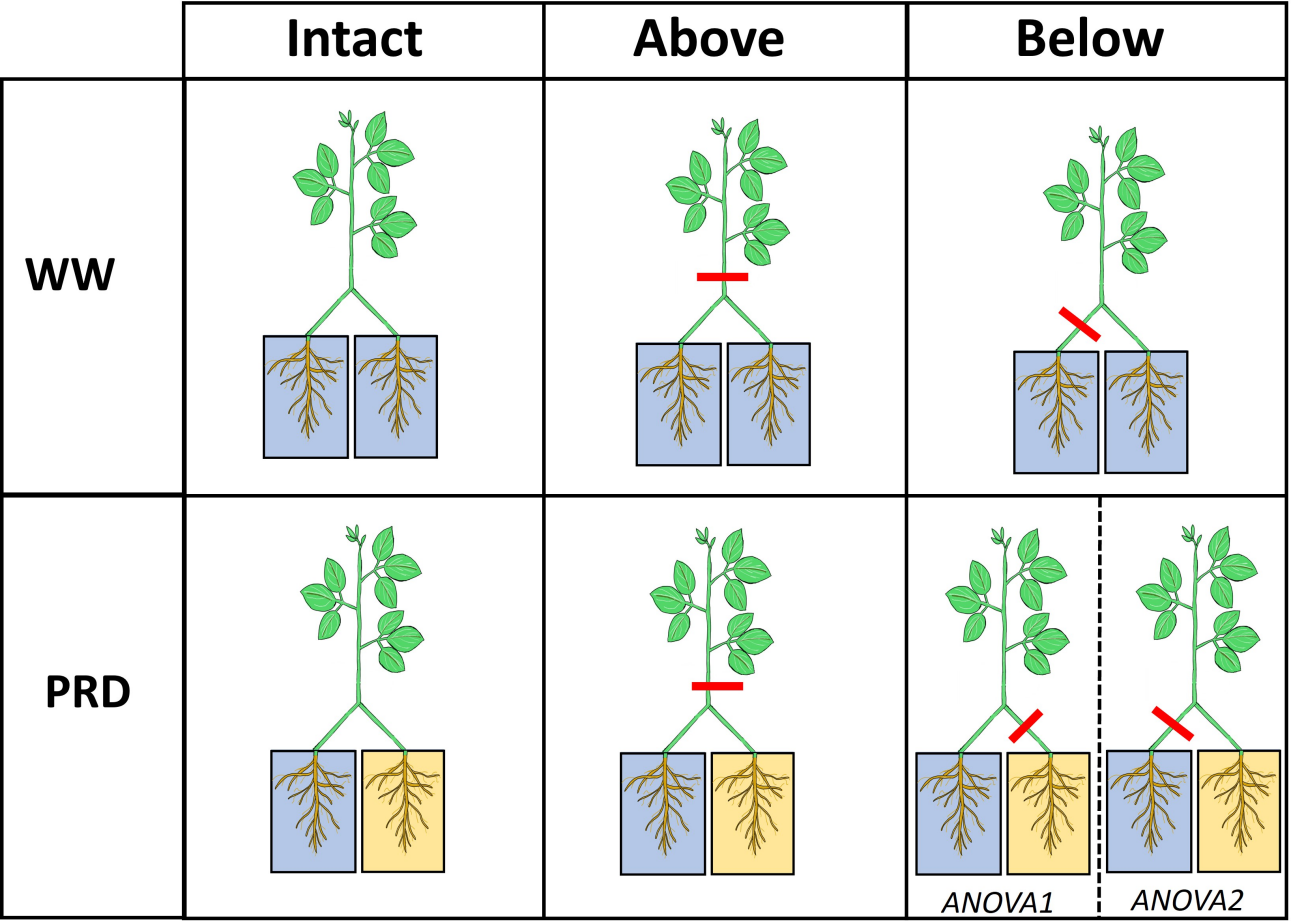
Schematic representation of the experimental treatments, comprising two irrigation treatments (WW: Well‐watered, PRD: Partial rootzone drying) and three girdling treatments (Intact: no girdling, Above: Girdled above the graft union, Below: Girdled one of the two stems below the graft union) applied to “two‐root, one‐shoot” (see Material and methods for further details) grafted soybean seedlings. For the PRD x Below combination, girdling was applied to the stem above the dry side (included in the analysis termed ANOVA1) or wet side (ANOVA 2). Blue and yellow columns represent the well‐watered and droughted sides (in PRD combinations), respectively. Red lines represent the location of the girdling: Intact (no girdling), and above or below (for WW plants) the graft union, on either the wet or the dry side of PRD plants.

To assess differential accumulation and export of ABA and other hormones between wet and dry parts of the rootzone in plants exposed to partial rootzone drying, phytohormone synthesis and redistribution within the plant need to be considered. Grafting a single scion onto two root systems of plants grown in two separate pots allowed phytohormone export from each root system to be measured (Dodd 2007). Interrupting phloem flow to both or either root system of such plants by surgically girdling the stem (Castro et al. 2019) above the graft union or one of the two basal stems below the graft union could distinguish the importance of basipetal hormone transport (Figure 1). In soybean (*Glycine max*), surgical girdling interrupts phloem shoot‐to‐root communication of multiple hormones, including ABA transport (Castro‐Valdecantos et al. 2021). Root tissue ABA accumulation and root xylem sap concentration of different phytohormones were assessed with combinations of heterogeneous and homogenous soil moisture and girdling different parts of the root zone. We tested the following hypotheses: (1) Compared to intact (non‐girdled) plants, interrupting phloem transport to one side of the root system would increase root ABA accumulation and export to shoots from the non‐interrupted part, as shoot‐sourced ABA flow is redirected to the other side of the root system. (2) Interrupting phloem ABA transport to both sides of the root zone would decrease overall root ABA accumulation and export to shoots (3) Phytohormones other than ABA known to be transported via phloem sap will follow a similar pattern as ABA.

## 2 MATERIALS AND METHODS

### 2.1 Plant materials and experimental design

Soybean seeds (*Glycine max* L. Merr. cv. Siverka) were sown in cylindric plastic pots that were customised to tightly fit inside a Scholander‐type pressure chamber (Soil Moisture Equipment Crop.), and filled with an organic loam (John Innes No. 2, J. Arthur Bowers). The pots were 6.5 cm in diameter and 23 cm in length (762 cm^3^ in volume), with the bottom made of a stainless‐steel mesh (0.7 mm aperture) to allow drainage. Seeds were gently placed 3 cm below the soil surface, covered with more substrate, and moistened. Plants were grown in a naturally lit greenhouse, supplemented with additional lighting when necessary to provide an average PPFD of 1000–1200 μmol m^−2^ s^−1^ at canopy height and 12 h of photoperiod. Greenhouse conditions were 27/16 ± 2/1°C daytime/nighttime temperature and a relative humidity of 30–40%. Each environmental parameter was recorded hourly in the centre of the glasshouse using a Hortimax Ektron II sensor (Pijnacker). All plants were watered to drained capacity at 16:00 h daily. A commercial liquid fertilizer Miracle‐Gro (24:8:16, N:P:K) was applied once (according to the manufacturer's instructions of 15 mL in 4.5 L of water) to the plants (each plant received 30 mL of the final solution) at the appearance of the first trifoliate leaf. Plants were grown under well‐watered conditions until the third trifoliate leaf started to develop.

To produce “two‐root, one shoot” grafted soybean plants, two pots were held together by rubber bands and the shoots of each plant were removed at the first internode, between the cotyledon scars (cotyledons had naturally abscised) and the first unifoliate leaf, with cuts at a 60° angle. The two stumps were bound with parafilm to form an inverted ‘Y' junction, into which a shoot apex was placed and bound with parafilm, as previously described (Dodd 2007). Each grafted plant was placed individually in a plastic bag to retain moisture for 10–12 days to allow the graft union to heal. After this establishment period, the plastic bags were removed for short intervals (increasing by 1–2 h each day) over a week to harden the plants. Unsuccessful grafts and plants with chlorotic graft unions were discarded to ensure both roots were connected to the shoot part. Four batches of plants, comprising 21 grafted plants per batch, were produced as described above at 7‐day intervals to ensure sufficient material for sequential harvests during the soil drying experiment.

When the third trifoliate leaf above the graft union had completely expanded, plants were randomized into 2 irrigation groups: well‐watered (WW) and PRD. Well‐watered plants were irrigated daily by replacing evapotranspirational losses determined gravimetrically, with water evenly distributed to both parts of the root system. Water was withheld from one column of the PRD plants during the experiment, and daily evapotranspirational losses were supplied to the other column. For each irrigation treatment, plants were randomly assigned to a different girdling treatment, as described below. Girdling was achieved surgically (at 10:00 h on Day 0) by excising 10 mm of phloem tissue (with a sharp razor blade) at either the middle of one of the two stem segments between the cotyledonary node and the graft union (below the graft union) or above the graft union. For PRD plants, girdling below the graft union was applied to either the stem with wet or dry roots, while for WW, girdling below the junction was randomly applied to one of the two stems. Thus, seven girdling x irrigation combinations were imposed (Figure 1). Measurements occurred 1, 3 and 5 days after girdling in the middle of the photoperiod. Each experiment measured one plant per girdling x irrigation treatment combination on these days, with 4 experiments repeated sequentially to allow 4 plants per combination in the entire study.

### 2.2 Measurements

Soil moisture status of each column was monitored to calculate root water uptake (RWU) as described previously (Puértolas et al., 2013), with some modifications. Briefly, two pairs of 6 mm diameter holes (2 cm apart horizontally) were punched in the pot wall on opposite sides of the pot, at 6 cm from the base and the top of the soil column, respectively (thus 11 cm apart vertically). On each pair of holes, two rods of a soil moisture capacitance probe (Model SM‐200; Delta‐T) connected to a datalogger (GP1, Delta‐T) were inserted to record soil volumetric water content every 5 minutes in each of the two (upper and lower) probe positions. Before measurements, the pot surface and the base were covered with aluminium foil to reduce soil evaporation and drainage, then the plant was weighed, and the soil moisture probes were inserted. Ninety minutes later, stomatal conductance (g_s_) was measured as described below, the sensors were removed, and the pot weighed. The total RWU of the whole plant was calculated gravimetrically, while the fraction of RWU from each column was calculated from the rate of change of soil volumetric water content during the time. After de‐topping the plant to collect sap (see below), leaf area (LA) was measured with a leaf area meter (Li‐3100C, LI‐COR). The whole plant transpiration rate (E) and E from each column (E_side_) were calculated from RWU (converted to molar unit) by dividing it by LA.

Stomatal conductance (g_s_) was measured on the central leaflet of the third trifoliate leaf with a porometer (Model AP4, Delta‐T Devices). Two measurements were sequentially made on each plant and averaged. After measuring g_s_, the entire leaf was excised and inserted in a pressure chamber (3000 Series Plant Water Status Console, Soil Moisture Equipment Corp.) to determine leaf water potential (Ψ_leaf_). The measured leaf was inserted in an Eppendorf vial, which was snap frozen in liquid nitrogen. After measuring Ψ_leaf_, the plant was de‐topped by severing the two stems below the graft union. Each of the two pots was simultaneously sealed in each of two pressure chambers (two operators working together) with sufficient stem protruding, including the remaining girdled tissue for girdled stems. After inserting the stem, the root system was pressurised to measure root water potential (Ψ_root_) and then overpressurised at 0.02 MPa intervals. At each step above the balancing pressure, sap was collected for 30 s in an Eppendorf tube and weighed to calculate the sap flow rate. The pressure was increased until the sap flow rate matched the estimated RWU of the measured root system (Else et al., 1995), which ranged between 0.1 and 1.0 μL s^−1^. At this moment, sufficient sap (50–100 μL) was collected, frozen in liquid nitrogen, and stored at −80°C to subsequently determine phytohormones.

Finally, root tissue samples (80–100 mg dry weight) were collected from the middle of the pot since, presumably, this zone presents average values between the lowest root ABA in the bottom and highest in the uppermost layer, coinciding with the highest and lowest soil water content, respectively, as observed in Puértolas et al. (2013). Samples were briefly washed (to remove adhering soil debris) and then frozen in liquid nitrogen. Plant tissues were stored at −80°C for further analysis. After collecting root tissue, the entire soil volume was removed from the pot, weighed, and then placed in a drying oven until constant weight to calculate gravimetric soil water content (θ_g_) with the following formula:

$$\text{Soil Water Content}\;\left(\theta_{g}\right)=\left(\text{Fresh soil weight}–\mathrm{Dry}\;\text{soil weight}\right)\;/\mathrm{Dry}\;\text{soil weight}$$

Soil water potential was calculated using the equation derived from a soil moisture release curve performed on the same type of soil as the one used in the current experiment (Castro‐Valdecantos et al. 2021).

### 2.3 Phytohormone analyses

Only ABA was determined in plant tissues using a radioimmunoassay using the monoclonal antibody MAC252 (Quarrie et al. 1988). Samples were lyophilized and finely ground. Deionized water was added (1:50 and 1:30 weight ratio for leaves and roots, respectively), the sample was incubated on a shaker at 4°C overnight, then centrifuged to collect the aqueous extract.

For root xylem sap, phytohormones including cytokinins (*trans*‐zeatin, tZ, zeatin riboside, ZR, and isopentenyl adenine, iP), gibberellins (GA_1_, GA_3_, and GA_4_), indole‐3‐acetic acid (IAA), abscisic acid (ABA), salicylic acid (SA), jasmonic acid (JA), and the ethylene precursor 1‐aminocyclopropane‐1‐carboxylic acid (ACC) were analysed according to (Albacete et al. 2008) with some modifications. Then, 10 μL of internal standard mix, comprising deuterated phytohormones ([^2^H_5_]tZ, [^2^H_5_]tZR, [^2^H_6_]iP, [^2^H_2_]GA_1_, [^2^H_2_]GA_3_, [^2^H_2_]GA_4_, [^2^H_5_]IAA, [^2^H_6_]ABA, [^2^H_4_]SA, [^2^H_6_]JA, [^2^H_4_]ACC, Olchemim Ltd., Olomouc, Czech Republic) at 1 μg·mL^−1^, were added to the extraction homogenate, and the samples were extracted at 4°C and shaking for 30 min. After the initial extraction, solids were separated by centrifugation (20,000 g) for 15 min and extracted again for 30 min at 4°C and shaken in an additional 1 mL of the same extraction solution. The pooled supernatants were passed through Sep‐Pak Plus C18 cartridges (SepPak Plus, Waters, USA) to remove interfering lipids and plant pigments and evaporated at 40°C under a vacuum to near dryness. The residue was dissolved in 0.5 mL methanol/water (20/80, v/v) in an ultrasonic bath. Xylem sap was filtered through 13 mm diameter Millex filters with 0.22 μm pore diameter nylon membrane (Millipore).

Ten μL of filtered extract were injected into a U‐HPLC‐HRMS system comprising an Accela Series U‐HPLC (ThermoFisher Scientific) coupled to an Exactive mass spectrometer (ThermoFisher Scientific) using a heated electrospray ionisation (HESI) interface. Xcalibur software version 2.2 (ThermoFisher Scientific) was used to obtain mass spectra. To quantify each hormone, calibration curves were constructed for each analysed component (1, 10, 50, and 100 μg L^−1^) and corrected for 10 μg L^−1^ deuterated internal standards. Recovery percentages ranged between 92 and 95%.

Root xylem sap concentrations of IAA, salicylic acid, cytokinins and the gibberellin GA_1_ were typically below the detection range and, thus, were excluded from analyses. For the detected phytohormones, delivery rate (amount of hormone per leaf area and time (Yong et al. 2000) from each side of the root system was calculated by multiplying the phytohormone concentration (expressed in pg. μl^−1^) by E_side_ (converted to μL m^−2^ s^−1^, resulting in pg. m^−2^ s^−1^). Total delivery to the shoot was calculated as the sum of deliveries from both parts of the root system.

### 2.4 Statistical analyses

Due to constraints imposed by the hypotheses tested, the two girdling treatments below the graft union in PRD plants (girdling the irrigated side, girdling the dry side) only had a single counterpart in the WW treatment (girdling the irrigated side). Thus, two different analyses of variance (ANOVA), each of a 2 × 3 factorial (*Irrigation* × *Girdling*) design, occurred. To compare the effect of girdling, PRD and its interaction on the dry side of PRD plants, ANOVA 1 excluded PRD plants girdled below the graft union of the wet side. To assess these effects on the wet side, ANOVA 2 excluded PRD plants girdled below the graft union on the dry side. For each ANOVA, three treatments were considered for the *Girdling* factor (no girdling, girdling above the graft union, and girdling below the graft union) and two for the *Irrigation* factor (WW and PRD).

For those variables associated with a particular side of the root (θ_g_, Ψ_root_, E_side_, [ABA]_root_, and xylem sap concentration and delivery of the detected phytohormones, i.e., ABA, JA, ACC, GA_3_ and GA_4_), firstly, a repeated measure ANOVA tested the hypothesis that significant differences between sides occurred only within PRD treatments. These repeated measures ANOVAs considered *Side* as within‐subject effect and Irrigation (WW and PRD), *Girdling* (Intact, Above, Below) and *Days* of withholding water (1, 3, 5) as between‐subject factors. For those variables where both *Side*, *Irrigation*, and *Side* × *Irrigation* interaction were significant (all variables except xylem GA_3_ and GA_4_ concentration and delivery), a separate 3‐way‐ANOVA (*Irrigation*, *Girdling* and *Day* of drought treatment) was conducted for each side. Those variables not associated with side (E, g_s_, Ψ_leaf_, [ABA]_leaf_, xylem phytohormone delivery rates) also received 3‐way‐ANOVA.

## 3 RESULTS

**FIGURE 2:**
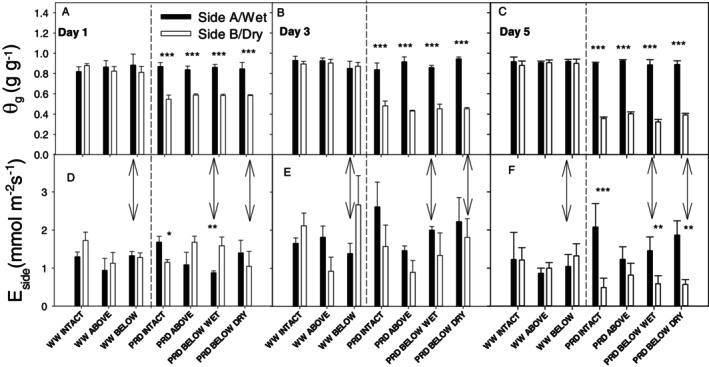
Soil gravimetric water content (θ_g_) of (A‐C) and transpiration rate (E_side_) from (D‐F) both sides of the root‐zone of “two‐root, one‐shoot” (see Material and methods for further details) grafted soybean seedlings for the different combinations of irrigation (WW = well‐watered; PRD = partial root‐zone drying) and girdling position (intact = no girdling; Above = above the graft union; Below = below the graft union; Wet = girdled on the wet side; Dry = girdled on the dry side) after 1 (A), 3 (B) and 5 (C) days of withholding water. Black bars = Side A (in WW) or wet side (in PRD). White bars = Side B (in WW) or dry side (in PRD). Double‐headed arrows mark for both panels the girdled side in treatments girdled below the graft union and the vertical dashed lines separate in each panel WW from PRD treatments. Asterisks denote statistical differences between sides (*: 0.01 < *p* < 0.05; **: 0.001 < *p* < 0.01; ***: *p* < 0.001); *n* = 3–4 (means + s.e.). No girdling × irrigation interaction was observed for either of the two sides (Table S1).

Soil gravimetric water content and water potential were high (θ_g_ ~ 0.90 g g^−1^; Ψ_soil_ > ‐1 kPa) and consistently similar over the five days of the experiment in both sides of WW and the wet side of PRD plants (Figure 2A–C). Girdling did not significantly affect θ_g_ of wet sides (Table S1). Withholding water from the dry side of PRD plants decreased soil moisture after just one day (θ_g_ ~ 0.60 g g^−1^; Ψ_soil_ = −10 kPa; Figure 2A), which continued to decrease during the following four days to ~0.35 g g^−1^ (Ψ_soil_ = −15 kPa; Figure 2B, C). In general, transpiration fluxes from the dry side of PRD plants (E_dry_) only significantly differed from that from the wet side (E_wet_) on day 5, with a maximum of a 4‐fold higher E_wet_ in PRD intact plants, and no significant difference in PRD plants girdled above the union (Figure 2D, E, F). Thus, soil moisture decreased in the dry side of PRD plants throughout the experiment independently of girdling but only affected transpiration fluxes from the dry side of PRD plants at the end.

**FIGURE 3:**
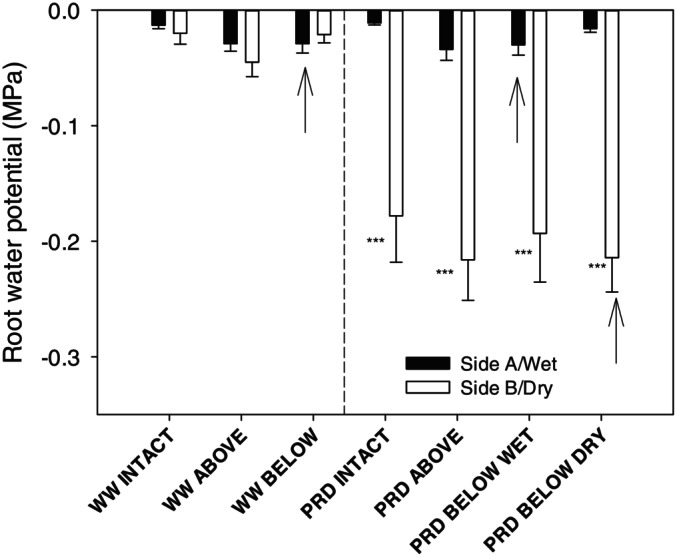
Root water potential of both sides of the rootzone of “two‐root, one‐shoot” (see Material and methods for further details) grafted soybean seedlings for the different treatment combinations of irrigation (WW = well watered; PRD = partial rootzone drying) and girdling positions (intact = no girdling; Above = above the graft union; Below wet = below the graft union on the wet side; Below dry = on the dry side). Black bars = Side A (in WW) or wet side (in PRD). White bars = Side B (in WW) or dry side (in PRD). Arrows mark the girdled side in treatments girdled below the graft union and the vertical dashed line separates WW from PRD treatments. Asterisks denote statistical differences between sides (***p < 0.001). Day effect was significant for the dry side (Table S1), as root water potential slightly decreased with time (data not shown), However, for clarity data is pooled across the three measurement days. *n* = 9–10 (means + s.e.). No girdling x irrigation interaction was observed for any of the two sides (Table S1).

Root water potential followed a similar trend as soil water potential, with high values (Ψ_root_ ~ −0.02 MPa) for WW and wet roots of PRD plants (Figure 3), but lower Ψ_root_ of the dry side of PRD (~ − 0.20 MPa) plants. Girdling significantly increased Ψ_root_ in WW and in wet roots of PRD (Table S1), but this effect was very small (~ − 0.02 MPa) compared to intact roots. Girdling did not significantly affect Ψ_root_ of the dry side of PRD plants. PRD significantly decreased leaf water potential (Ψ_leaf_) by almost 0.1 MPa (−0.8 vs. −0.7 MPa for PRD and well‐watered plants, respectively), but girdling did not (Table S2). Thus, PRD decreased both Ψ_root_ in the dry roots and Ψ_leaf_, while girdling only slightly increased Ψ_root_ in the wet roots of PRD.

**FIGURE 4:**
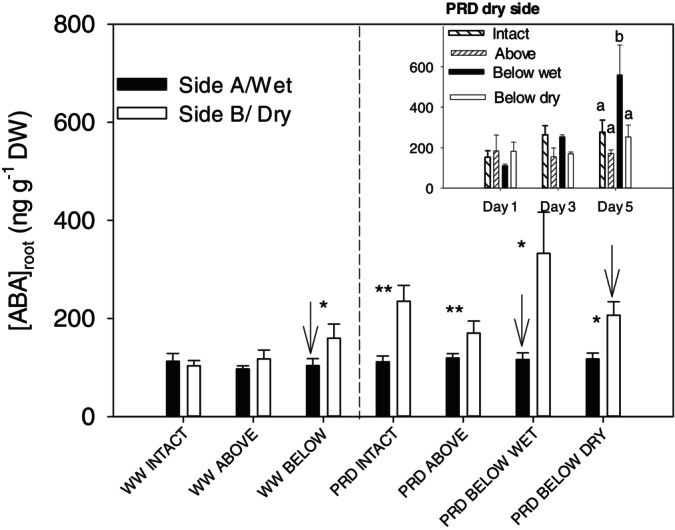
Root ABA accumulation ([ABA]_root_) in both sides of the root‐zone of “two‐root, one‐shoot” (see Material and methods for further details) grafted soybean seedlings for the different treatment combinations of irrigation (WW = well watered; PRD = partial root‐zone drying) and girdling positions (intact = no girdling; Above = above the graft union; Below wet = below the graft union on the wet side; Below dry = on the dry side). Black bars = Side A (in WW) or wet side (in PRD). White bars = Side B (in WW) or dry side (in PRD), (n = 3–4; mean + s.e.). Arrows mark the girdled side in treatments girdled below the graft union. In the main panel, data is pooled across the three measurement days. n = 9–10 (mean + s.e.). Since ANOVA2 (PRD below wet included) showed a significant effect of Day and Day x girdling for the dry side in contrast with ANOVA 1 (with PRD below dry included), the insert shows how [ABA]_root_ changed in the dry side of the PRD treatments during the experiment (right‐to‐left ascending pattern: Intact; left‐to‐right ascending pattern: Girdling above the graft union; Black: girdling below the graft union in the wet side; White: girdling below the graft union in the dry side). For the main panel, asterisks denote statistical differences between sides (*0.01 < *p* < 0.05; **p < 0.01). For the insert, different letters denote statistical differences between means within a measurement date; no letters denoting no statistical differences (Tukey, *p* < 0.05).

Root ABA concentration ([ABA]_root_) was higher in the dry side of PRD treatments than in other soil compartments, regardless of girdling (Figure 4, Table S1). However, girdling the wet side below the graft union in PRD induced higher [ABA]_root_ in the dry, intact side than the rest of the girdling treatments (40, 95 and 60% for intact, girdled above the graft union and girdled in the dry side respectively). Moreover, this higher root ABA concentration in the dry side of plants girdled on the wet side increased during the experiment (Figure 4, insert). In contrast, girdling the dry side below the graft union did not increase ABA accumulation in the wet intact side. Well‐watered plants also showed this effect, as girdling below (but not above) the graft union increased [ABA]_root_ in the other root (significant girdling effect but no girdling × irrigation interaction for the dry side in ANOVA2; Table S1). Thus, in general, phloem transport interruption to one side of the root zone increased ABA concentration on the other side compared to intact plants, except when interrupting to the dry side of PRD plants, as ABA in the well‐watered side remained similar to intact plants.

**FIGURE 5:**
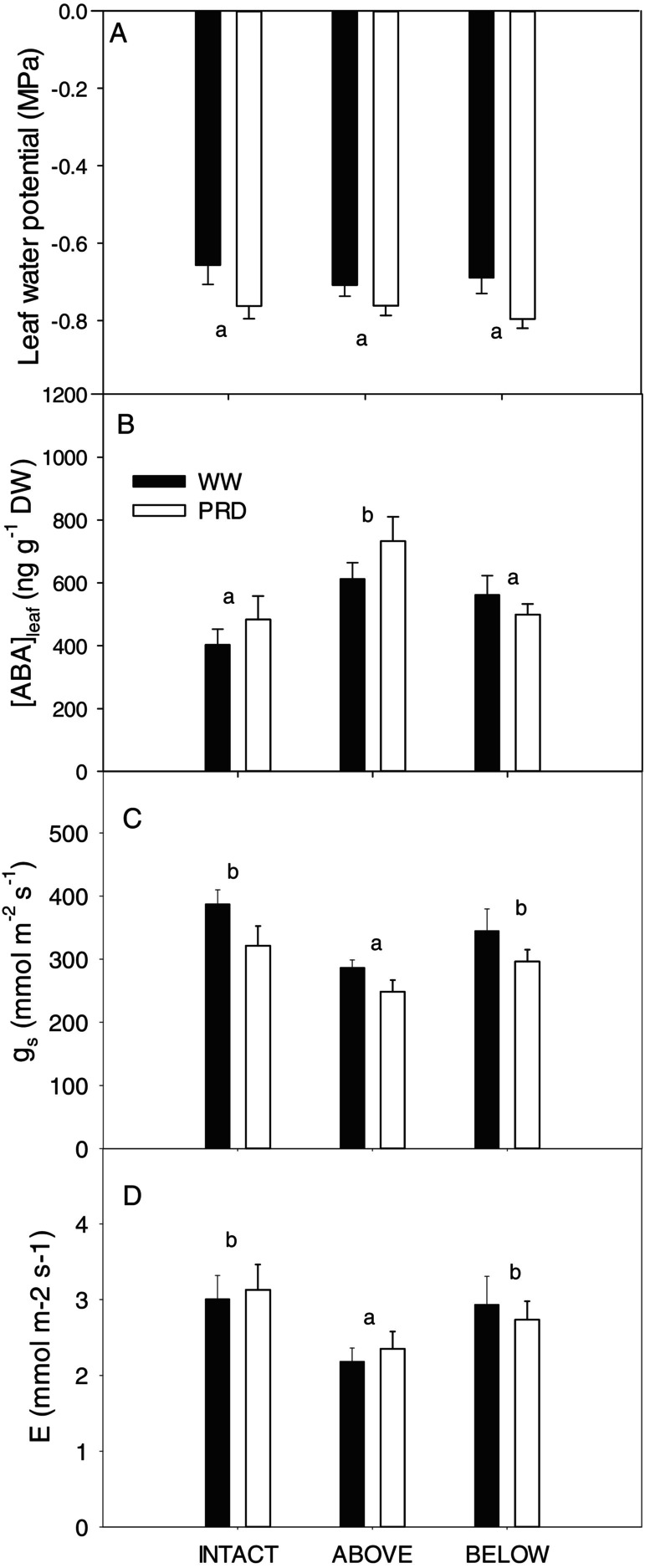
Leaf water potential (A), ABA concentration ([ABA]_leaf_, B), stomatal conductance (g_s_, C), and transpiration rate (E, D) of “two‐root, one‐shoot” (see Material and methods for further details) grafted soybean seedlings for the different treatment girdling positions (Intact = no girdling; Above = above the graft union; Below = below the graft union, with Black bars = WW and white bars = PRD). PRD Below data pooled across the two girdling positions (wet side and dry side; n = 9‐for intact, above, and WW above, 18 for PRD below; mean + S.E.). Different letters denote statistical differences between girdling position averages (Tukey, *p* < 0.05). Irrigation was significant for leaf water potential and g_s_ but not for [ABA]_leaf_, while irrigation × girdling interaction was not significant (Table S2).

Foliar ABA concentration [ABA]_leaf_ did not differ between WW and PRD plants (Table S2) but increased by 50% and 30% in plants girdled above the graft union or just one side below the graft union, respectively, compared to intact plants (Figure 5A). Stomatal conductance was highest in WW plants (Figure 5B) and was higher in intact plants than those girdled on one side only or above the graft union (by 10% and 30% respectively). Transpiration rate followed the same pattern regarding girdling, but irrigation treatments did not differ (Figure 5D, Supplementary Table 2). Therefore, girdling increased leaf ABA and decreased g_s_ proportionally to the fraction of the root whose phloem supply was interrupted by girdling.

**FIGURE 6:**
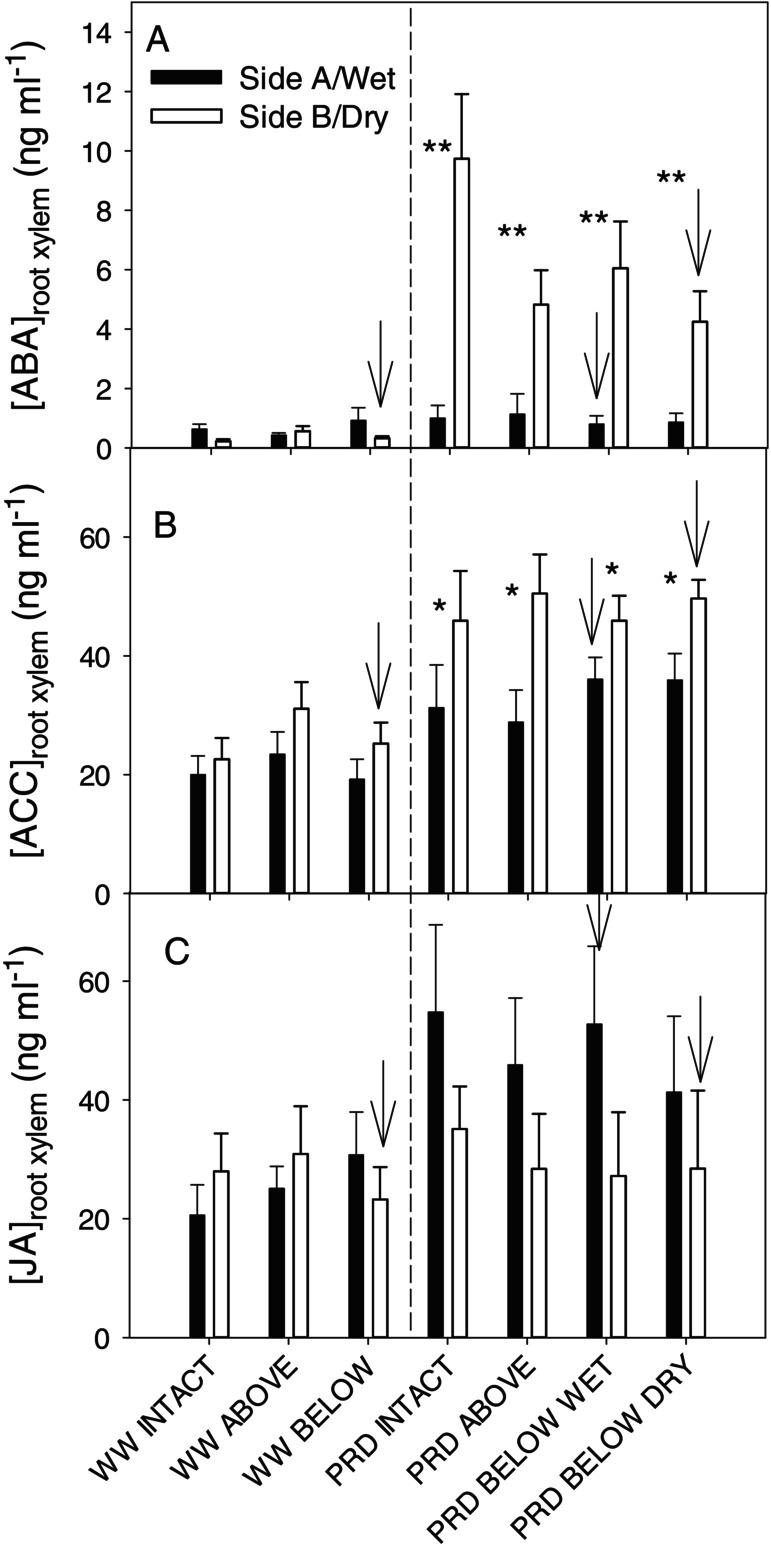
Root xylem sap concentration of the phytohormones that showed a significant *Side* x *Irrigation* treatment interaction, specifically Abscisic acid, ABA (A), Jasmonic acid, JA (B) and 1‐aminocyclopropane‐1‐carboxylic acid, ACC (C) of “two‐root, one‐shoot” (see Material and methods for further details) grafted soybean seedlings, collected from either side of the root‐zone for the different treatment combinations of irrigation (WW = well‐watered; PRD = partial rootzone drying) and girdling positions (intact = no girdling; Above = above the graft union; Below wet = below the graft union on the wet side; Below dry = on the dry side). Black bars = Side A (in WW) or wet side (in PRD). White bars = Side B (in WW) or dry side (in PRD). Arrows mark the girdled side in treatments girdled below the graft union and the vertical dashed line separates WW from PRD treatments. Asterisks denote statistical differences between sides (***p < 0.001). Data pooled across the three measurement days. *n* = 8–10 (mean + s.e.)

Neither irrigation nor girdling affected root xylem sap concentrations of the two gibberellins measured (data not shown). Girdling did not affect the concentrations of the other detected hormones: ABA, JA and ACC (Table S3). However, xylem sap ABA concentration from the dry roots of PRD plants was significantly higher (4 to 10‐fold, Figure 6A) than from the wet roots (with PRD wet or WW roots having similarly low ABA concentrations). PRD also significantly increased xylem sap ACC concentration from drying roots (by 80%), and from irrigated roots (by 60%) compared to WW plants (Table S3, Figure 6B). Unlike ABA and ACC, sap collected from irrigated roots (but not dry roots) of PRD plants had higher (90%) JA concentration than collected from roots of WW plants (Figure 6C). While girdling did not affect xylem sap phytohormone concentrations, PRD increased xylem ABA concentrations from drying roots, JA concentrations from wet roots, and ACC concentrations from both sides of the root system.

**FIGURE 7:**
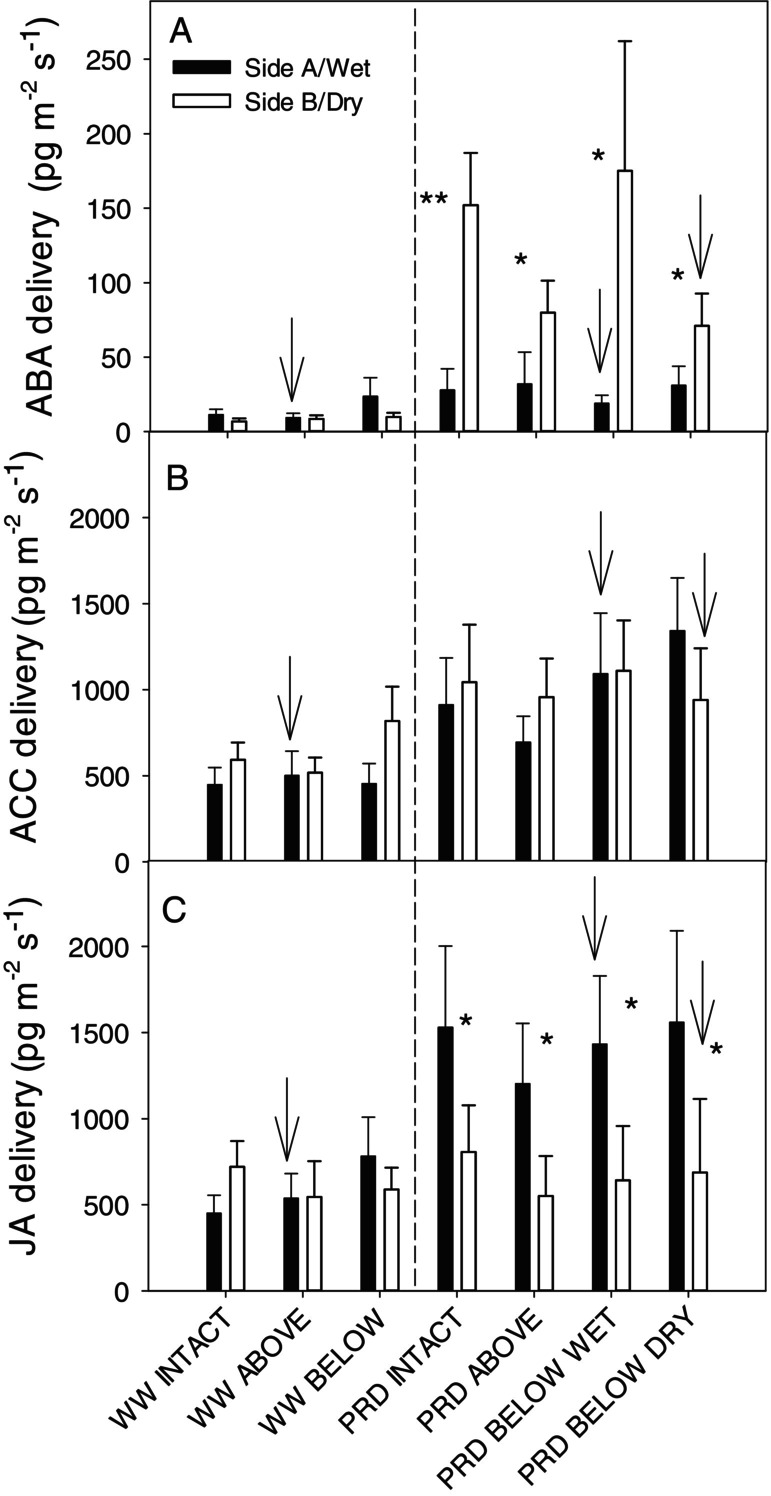
Delivery rate in xylem sap of Abscisic acid, ABA (A), Jasmonic acid, JA (B) and 1‐aminocyclopropane‐1‐carboxylic acid, ACC (C) from either side of the rootzone of “two‐root, one‐shoot” (see Material and methods for further details) grafted soybean seedlings, for the different treatment combinations of irrigation (WW = well‐watered; PRD = partial root‐zone drying) and girdling positions (intact = no girdling; Above = above the grafting union; Below wet = below the grafting union on the wet side; Below dry = on the dry side), as these three phytohormones showed a significant side × irrigation treatment interaction. Black bars = Side A (in WW) or wet side (in PRD). White bars = Side B (in WW) or dry side (in PRD). Arrows mark the girdled side in treatments girdled below the grafting union and the vertical dashed line separates WW from PRD treatments. Asterisks denote statistical differences between sides (***p < 0.001). Data pooled across the three measurement days. n = 8–10 (mean + S.E.)

Since differences in E_side_ in PRD plants were only significant on Day 5, in general, phytohormone deliveries (concentration x transpiration rate) were very similar to those described for phytohormone concentrations. ABA delivery from the root system of PRD plants was much greater (6‐fold) than well‐watered plants. PRD significantly increased ACC delivery by 85%. While only the dry side exported more ABA than either side of WW plants, both parts of the rootzone contributed to greater ACC delivery (Figure 7). PRD tended to increase JA delivery (although not significantly, *p* < 0.08) due to export from wet roots (Table S4, data not shown). Thus, hormone export from roots of PRD plants was greater than from WW plants.

## 4 DISCUSSION

Whether sap fluxes from wet and dry parts of the root system regulate root‐to‐shoot phytohormone signalling during PRD is not clear, as few experiments measured those fluxes (Dodd et al. 2008b). As the shoot is considered to regulate root ABA (Manzi et al. 2015; McAdam et al. 2016) and jasmonate (Luo et al. 2019) accumulation, interrupting phloem transport to different parts of the root system (Figure 1) was expected to cause differential phytohormone accumulation in, and thus export from, roots. Instead, differential root export of several compounds (ABA, ACC and JA) between wet and dry parts of the rootzone occurred independently of the site at which phloem transport was interrupted (Figure 6). This focused attention on the potential synthesis of these compounds in roots exposed to different soil moisture, with genes encoding enzymes that catalyse rate‐limiting steps of ABA (9‐cis‐epoxycarotenoid dioxygenase – NCED – Thompson et al. 2000), ACC (ACC synthase – Olson et al. 1995) and JA (lipoxygenase ‐ Bell and Mullet 1991) synthesis all expressed in roots. How the plant integrates such root‐sourced *versus* shoot‐sourced long‐distance phytohormone signalling seems important in the agronomic context of heterogenous soil moisture increasing crop yields (Dodd 2009) even in soybean (Zhou et al. 2024).

Even when stem girdling interrupted basipetal ABA transport from shoot to roots, partial rootzone drying caused ABA accumulation exclusively in drying roots (Figure 4, 6; Table S1). While consistent with patterns of ABA accumulation in osmotically stressed (PEG‐6000) cotton roots irrespective of girdling (Luo et al. 2019), this differential accumulation could result from either active ABA biosynthesis or passive accumulation due to decreased root ABA export in the xylem (Shi et al. 2015). Similar root water uptake between dry and wet roots (Figure 2), causing greater xylem ABA concentration and delivery to the shoots in PRD than in WW plants, makes the latter argument unlikely. Indeed, dry roots contributed mostly to increased ABA delivery (Figure 7). Root dehydration upregulates root expression of ABA biosynthesis genes (Speirs et al. 2013; Manzi et al. 2016), thus lowering root water potential (Figure 3) likely stimulated ABA biosynthesis in the dry part of the rootzone under PRD. Although root water status decreased by no more than −0.2 MPa (Figure 3), these changes substantially increased ABA accumulation in detached roots (Lachno and Baker, 1986), with a linear relationship between root water potential and xylem ABA concentration (Puértolas et al. 2013). While girdling previously prevented (Manzi et al. 2015) or restricted (Castro et al. 2019) root ABA accumulation when the entire rootzone was dried, roots of PRD plants in drying soil accumulated ABA despite girdling. Upregulation of ABA biosynthesis genes in response to decreased Ψ_root_ (Speirs et al. 2013; Zdunek‐Zastocka and Sobczak 2013) in the dry rootzone offers the simplest explanation for this effect.

Similarly, dehydration stimulated ABA synthesis in detached roots (Cornish and Zeevaart 1985; Simonneau et al. 1998). Root ABA synthesis requires carotenoids, but their abundance is unlikely to be limiting (Manzi et al. 2016), as they are substrates of the isoprenoid synthesis MEP pathway and can be synthesised in root plastids (Ruiz‐Sola et al. 2014). However, girdling also interrupts carbohydrate import, rapidly depleting carbohydrates in this heterotrophic tissue (Li et al. 2003). Declining energy supply should impair root primary metabolism, thereby decreasing the availability of isoprenoid precursors needed for carotenoid synthesis along with the energy necessary for that synthesis (Banerjee and Sharkey 2014). This may explain why girdling prevented soil root ABA accumulation with repeating soil drying (Manzi et al. 2015) but contradicts differential ABA accumulation in dry roots of girdled PRD plants (Figure 4).

Girdling effectively blocked phloem ABA transport to the roots, causing additional shoot ABA accumulation, thereby exacerbating stomatal closure (Figure 5C) as previously observed (Setter et al. 1980). Furthermore, girdling redirected ABA to drying roots when the phloem flow to the wet side of PRD plants was interrupted (Figure 4). Notably, this redirection did not occur (or did not affect root ABA concentration) when the dry side was girdled since root ABA concentration did not increase in the opposite wet side. While suggesting phloem ABA transport occurs preferentially towards drying roots, greater ABA accumulation on the intact side of WW plants when the other side was girdled disproves this. Why ABA does not accumulate on the wet side when the dry side is girdled is unclear.

Why ABA accumulated in drying roots under PRD despite phloem transport blockage (Figure 1), unlike with homogeneous soil drying (Manzi et al. 2015), requires explanation. More severe root water deficits with homogeneous soil drying may decrease ABA biosynthesis or increase ABA catabolism compared to those under PRD, restricting root ABA accumulation in the absence of leaf‐sourced ABA. Severe dehydration (e.g. transplanting bare‐rooted plants to dry perlite in Manzi et al. 2015) impaired root ABA accumulation (Zhang and Davies 1990; Kulkarni et al. 2000) and enhanced ABA catabolism (Ren et al., 2007). This may explain why roots cannot accumulate ABA when shoot‐to‐root ABA transport is impeded. However, exposing girdled soybean plants to similar or even milder levels of soil drying than those achieved here caused less root ABA accumulation than their intact counterparts (Castro et al. 2019; Castro‐Valdecantos et al. 2021). Therefore, the severity of soil drying alone may not entirely explain the lack of ABA accumulation.

Alternatively, PRD‐induced changes in other phytohormones could enhance ABA synthesis (or decrease catabolism) independent of girdling. PRD increased JA levels in xylem sap from the wet part of the root system compared to the dry part (Figure 6C), indicating either enhanced JA synthesis in wet roots or (unlikely as JA levels in drying roots were similar to WW plants) or decreased synthesis in drying roots. This response was unexpected, as whole soil drying also increased root JA (and ABA) levels in intact but not girdled plants (Castro‐Valdecantos et al. 2021). Moreover, applying polyethylene glycol (PEG) to half the root system of hydroponically grown cotton plants increased root JA‐Isoleucine conjugate (JA‐Ile) concentration on both sides, more so in PEG‐treated roots (Luo et al. 2019). As PEG treatment upregulated JA biosynthesis genes (*Gh0PR11*, *GhA0S6, GhLOX3*) in osmotically stressed roots but not non‐stressed roots, this suggests leaves were the source of root JA‐Ile accumulation. While PRD triggers JA export from wet roots (Figure 6C), the lack of JA increase in xylem sap from the dry side and its independence of stem girdling is difficult to reconcile with the increased JA gene expression in osmotically‐stressed roots and leaf‐sourced accumulation in non‐stressed roots (Luo et al. 2019). Although JA did not accumulate concomitantly with ABA (de Ollas et al. 2018) in the dry side of PRD plants (cf. Figure 6A, C), our sampling frequency may not have detected transient root JA accumulation (de Ollas and Dodd 2016). Irrespective of shoot‐to‐root transport, transient overwatering might increase JA export from the wet part of the root zone. Although soil moisture of WW plants and the irrigated side of PRD plants was similar when the sap was collected (Figure 1), the wet columns of PRD plants received twice the irrigation volume of WW columns with transient over‐irrigation potentially increasing root xylem sap JA concentration. Increased root JA‐Ile under low oxygen conditions is reportedly involved in restricting primary root growth (Shukla et al. 2020).

The PRD treatments also increased root xylem sap concentration of the ethylene precursor ACC from both sides, especially from drying roots (Figure 6B), as in PRD‐grown tomato in which soil drying increased root ACC levels, and re‐watering increased xylem ACC concentrations (Pérez‐Pérez et al. 2020). Low and very high soil moisture increases the foliar ethylene evolution of tomato (Fiebig 2014). Therefore, both dry and transient overwatering (as described above) could increase ACC export from both irrigated and dry roots. Similarly, PRD increased shoot ethylene evolution compared to both WW and homogeneous drying (Sobeih et al. 2004; Marino et al. 2017), suggesting increased ACC signalling in response to PRD. Girdling did not perturb root xylem ACC concentrations (Figure 6B), suggesting increased local ACC biosynthesis as with the other phytohormones discussed above. Attenuating root‐to‐shoot ACC signalling (Belimov et al. 2009) or restricting ACC conversion to ethylene (Sobeih et al. 2004) maintained leaf growth as the soil dried but did not affect stomatal responses. In citrus, suppressing ABA biosynthesis by treating roots with norflurazon prevented root ACC accumulation, suggesting ABA was needed to elicit ACC accumulation (Gómez‐Cadenas et al. 1996). Therefore, PRD enhanced ACC export from both sides of the roots by drying one part of the root system and transiently overwatering the other.

Changes in root phytohormonal (especially ABA) export that induce partial stomatal closure without decreasing leaf water potential are believed to increase water use efficiency under PRD (Blackman and Davies 1985; Stoll et al. 2000). Although PRD decreased instantaneous stomatal conductance by less than 20% in intact plants (Figure 5C), whole plant transpiration did not decline (Figure 5D), regardless of girdling. Leaf water potential also decreased from −0.6 to −0.7 MPa (Figure 5A) concurrent with partial stomatal closure, as when plants of the same species and age were grown in the same pots and growing medium (Castro et al. 2019). However, unlike in roots, where a similar change in water potential induced ABA accumulation, this small decrease in leaf water potential did not stimulate foliar ABA accumulation (Figure 5B). While girdling‐induced increases in bulk foliar ABA concentration decreased both stomatal conductance and transpiration, leaf ABA concentration does not seem to mediate PRD‐induced stomatal closure, with xylem sap ABA concentration better explaining stomatal closure than bulk leaf ABA in this species (Liu et al. 2003) and others (Zhang and Davies 1990; Peuke 2016). Our experiment cannot resolve whether hydropassive mechanisms mediated by slightly decreased epidermal cell turgor (Buckley 2019) or enhanced xylem‐borne ABA (Zhang and Davies, 1990), ACC (Else et al. 1995), or JA (de Ollas et al., 2018) delivered to the epidermal apoplast cause PRD‐induced stomatal closure. While foliar ABA increments cannot explain this stomatal behaviour, decreased leaf water potential and/or increased root xylem sap phytohormone concentrations might.

In conclusion, partial rootzone drying increased the xylem export of three known root‐to‐shoot signals (ABA, ACC and JA) of soil drying. Interrupting phloem transport from the shoots did not influence root phytohormone export in the xylem, suggesting enhanced local biosynthesis and increased export. Different phytohormones varied in their soil moisture responses, as ABA increased from the dry root zone, JA increased from the wet root zone, and ACC increased from both parts of the root zone. Increased levels of these hormones with heterogeneous soil drying depended on differences in local root (Figure 3) and not shoot (Figure 5A) water status. PRD slightly decreased stomatal conductance compared to WW plants, which was not explained by increased foliar bulk ABA concentration but by decreased leaf water potential and/or enhanced phytohormone export from roots. Since root hormone accumulation regulates not only root‐to‐shoot signalling but also root function and growth, such responses likely contribute to greater yields of plants grown with partial rootzone drying.

## AUTHOR CONTRIBUTIONS

J.P.: Conception and design of the study, data acquisition and analysis and drafting the first version of the draft and revising subsequent versions, P. C.‐V.: Conception and design of the study, data acquisition and revising all versions of the draft. A.A: Data acquisition and revising a final version of the draft. I.C.D: Conception and design of the study and revising all versions of the draft.

## Supporting information


**Supplementary Table 1.** Results of the analyses of variance (*P*‐values) for plant variables measured in either part of the root zone (wet side and dry side). θ_g_ = soil gravimetric water content, RWU = Root water uptake. Ψ_root_ = root water potential. [ABA]_root_ = Abscísic acid concentration in the root tissues. Plants girdled in the wet side of the plant were excluded in *ANOVA 1*, while those girdled in the dry side were excluded from *ANOVA* 2. In bold, significant *P*‐values (*p* < 0.05)
**Supplementary Table 2.** Results of the analyses of variance (P‐values) for aboveground plant variables. g_s_ = stomatal conductance, RWU = Total Root water uptake (as the sum of the RWU from both sides of the rootzone). Ψ_leaf_ = leaf water potential. [ABA]_leaf_ = Abscísic acid concentration in the leaf tissues. Plants girdled in the wet side of the plant were excluded in *ANOVA 1*, while those girdled in the dry side were excluded from *ANOVA* 2. In bold, significant *P*‐values (*p* < 0.05)
**Supplementary Table 3.** Results of the analyses of variance (*P*‐values) for phytohormone concentration in root xylem sap ([Phytohormone]_xylem_) and its delivery to the shoot (Phytohormone_delivery_) measured in either part of the root zone (wet side and dry side). ABA = Abscísic acid. JA = Jasmonic acid. ACC = 1‐Aminocyclopropane‐1‐carboxylic acid. Plants girdled in the wet side of the plant were excluded in *ANOVA 1*, while those girdled in the dry side were excluded from *ANOVA* 2. In bold, significant *P*‐values (*p* < 0.05)
**Supplementary Table 4.** Results of the analyses of variance (*P*‐values) for total phytohormone delivery to the shoot (Phytohormone_delivery_). ABA = Abscísic acid. JA = Jasmonic acid. ACC = 1‐Aminocyclopropane‐1‐carboxylic acid. Plants girdled in the wet side of the plant were excluded in *ANOVA 1*, while those girdled in the dry side were excluded from *ANOVA* 2. In bold, significant *P*‐values (*p* < 0.05)

## Data Availability

All data supporting the findings of this study are available within the paper and within its supplementary materials published online. The data that supports this study will be shared upon reasonable request to the corresponding author.
