# Supplementary material for: Differential xylem phytohormone export from dry and wet roots during partial rootzone drying is independent of shoot‐to‐root transport in soybean
Source: Physiol Plant. 2025 Apr 29;177(3):e70252. doi: 10.1111/ppl.70252 (PMC12041630; doi:10.1111/ppl.70252)
Supplement: Supplementary file 1 — Supplementary Table 1. Results of the analyses of variance (P‐values) for plant variables measured in either part of the root zone (wet side and dry side). θg = soil gravimetric water content, RWU = Root water uptake. Ψroot = root water potential. [ABA]root = Abscísic acid concentration in the root tissues. Plants girdled in the wet side of the plant were excluded in ANOVA 1, while those girdled in the dry side were excluded from ANOVA 2. In bold, significant P‐values (p < 0.05) Supplementary Table 2. Results of the analyses of variance (P‐values) for aboveground plant variables. gs = stomatal conductance, RWU = Total Root water uptake (as the sum of the RWU from both sides of the rootzone). Ψleaf = leaf water potential. [ABA]leaf = Abscísic acid concentration in the leaf tissues. Plants girdled in the wet side of the plant were excluded in ANOVA 1, while those girdled in the dry side were excluded from ANOVA 2. In bold, significant P‐values (p < 0.05) Supplementary Table 3. Results of the analyses of variance (P‐values) for phytohormone concentration in root xylem sap ([Phytohormone]xylem) and its delivery to the shoot (Phytohormonedelivery) measured in either part of the root zone (wet side and dry side). ABA = Abscísic acid. JA = Jasmonic acid. ACC = 1‐Aminocyclopropane‐1‐carboxylic acid. Plants girdled in the wet side of the plant were excluded in ANOVA 1, while those girdled in the dry side were excluded from ANOVA 2. In bold, significant P‐values (p < 0.05) Supplementary Table 4. Results of the analyses of variance (P‐values) for total phytohormone delivery to the shoot (Phytohormonedelivery). ABA = Abscísic acid. JA = Jasmonic acid. ACC = 1‐Aminocyclopropane‐1‐carboxylic acid. Plants girdled in the wet side of the plant were excluded in ANOVA 1, while those girdled in the dry side were excluded from ANOVA 2. In bold, significant P‐values (p < 0.05) [file PPL-177-e70252-s001.docx]

**Supplementary Table 1.** Results of the analyses of variance (*P*-values) for plant variables measured in either part of the root zone (wet side and dry side). θ_g_= soil gravimetric water content, RWU= Root water uptake. Ψ_root_= root water potential. [ABA]_root_= Abscísic acid concentration in the root tissues. Plants girdled in the wet side of the plant were excluded in *ANOVA 1*, while those girdled in the dry side were excluded from *ANOVA* 2. In bold, significant *P*-values (*P*<0.05)

|  | | **θ_g_** | |  | **RWU** | |  | **Ψ_root_** | |  | **[ABA]_root_** | | | |  |
| --- | --- | --- | --- | --- | --- | --- | --- | --- | --- | --- | --- | --- | --- | --- | --- |
|  | | *P-value ANOVA1* | *P-Value ANOVA2* |  | *P-value ANOVA1* | *P-Value ANOVA2* |  | *P-value ANOVA1* | *P-Value ANOVA2* |  | *P-value ANOVA1* | | *P-Value ANOVA2* | |  |
| *Wet side* | |  |  |  |  |  |  |  |  |  |  |  |  |  |  |
| Irrigation (I) | | 0.79 | 0.54 |  | **0.03** | 0.08 |  | 0.53 | 0.82 |  | | 0.25 | | 0.26 | |
| Girdling (G) | | 0.82 | 0.73 |  | 0.11 | 0.06 |  | **0.02** | **0.02** |  | | 0.95 | | 0.95 | |
| Day (D) | | 0.09 | 0.13 |  | 0.07 | **0.02** |  | 0.42 | 0.30 |  | | 0.27 | | 0.60 | |
| I x G | | 0.89 | 0.94 |  | 0.35 | 0.28 |  | 0.37 | 0.89 |  | | 0.69 | | 0.64 | |
| I x D | | 0.99 | 0.83 |  | 0.60 | 0.49 |  | 0.78 | 0.73 |  | | 0.25 | | 0.31 | |
| G x D | | 0.97 | 0.77 |  | 0.99 | 0.99 |  | 0.74 | 0.69 |  | | 0.54 | | 0.67 | |
| I x G x D | | 0.31 | 0.66 |  | 0.86 | 0.72 |  | 0.88 | 0.63 |  | | 0.89 | | 0.16 | |
| *Dry side* | |  |  |  |  |  |  |  |  |  | |  | |  | |
| Irrigation (I) | | **<0.001** | **<0.001** |  | **0.05** | **0.03** |  | **<0.001** | **<0.001** |  | | **<0.001** | | **<0.001** | |
| Girdling (G) | | 0.89 | 0.52 |  | 0.10 | 0.13 |  | 0.44 | 0.46 |  | | 0.28 | | **0.02** | |
| Day (D) | | **0.004** | **0.001** |  | **<0.001** | **0.002** |  | **0.05** | **0.02** |  | | 0.44 | | **0.05** | |
| I x G | | 0.64 | 0.89 |  | 0.15 | 0.13 |  | 0.77 | 0.96 |  | | 0.21 | | 0.28 | |
| I x D | **<0.001** | | **<0.001** |  | 0.47 | 0.16 |  | **0.008** | **0.003** |  | | 0.20 | | **0.02** | |
| G x D | 0.69 | | 0.77 |  | **0.02** | 0.14 |  | 0.99 | 0.93 |  | | 0.54 | | 0.21 | |
| I x G x D | 0.54 | | 0.33 |  | 0.84 | 0.58 |  | 0.96 | 0.82 |  | | 0.60 | | **0.03** | |

**Supplementary Table 2.** Results of the analyses of variance (P-values) for aboveground plant variables. g_s_= stomatal conductance, RWU= Total Root water uptake (as the sum of the RWU from both sides of the rootzone). Ψ_leaf_= leaf water potential. [ABA]_leaf_= Abscísic acid concentration in the leaf tissues. Plants girdled in the wet side of the plant were excluded in *ANOVA 1*, while those girdled in the dry side were excluded from *ANOVA* 2. In bold, significant *P*-values (*P*<0.05)

|  | **g_s_** | |  | **RWU** | |  | **Ψ_leaf_** | |  | **[ABA]_leaf_** | | | |  |
| --- | --- | --- | --- | --- | --- | --- | --- | --- | --- | --- | --- | --- | --- | --- |
|  | *P-value ANOVA1* | *P-Value ANOVA2* |  | *P-value ANOVA1* | *P-Value ANOVA2* |  | *P-value ANOVA1* | *P-Value ANOVA2* |  | *P-value ANOVA1* | | *P-Value ANOVA2* | |  |
| *Wet side* |  |  |  |  |  |  |  |  |  |  |  |  |  |  |
| Irrigation (I) | **0.04** | **0.007** |  | 0.69 | 0.87 |  | **0.002** | **0.003** |  | | 0.39 | | 0.11 | |
| Girdling (G) | **0.006** | **0.004** |  | **0.008** | **0.008** |  | 0.39 | 0.42 |  | | **<0.001** | | **<0.001** | |
| Day (D) | **0.05** | **0.01** |  | **<0.001** | **<0.001** |  | **0.006** | **0.001** |  | | **<0.001** | | **<0.001** | |
| I x G | 0.79 | 0.81 |  | 0.74 | 0.48 |  | 0.67 | 0.58 |  | | 0.05 | | 0.50 | |
| I x D | 0.78 | 0.52 |  | 0.96 | 0.80 |  | 0.17 | 0.22 |  | | 0.56 | | 0.78 | |
| G x D | 0.96 | 0.77 |  | 0.25 | 0.43 |  | 0.63 | 0.73 |  | | 0.09 | | 0.14 | |
| I x G x D | 0.52 | 0.73 |  | 0.70 | 0.78 |  | 0.81 | 0.96 |  | | 0.35 | | 0.25 | |

**Supplementary Table 3.** Results of the analyses of variance (*P*-values) for phytohormone concentration in root xylem sap ([Phytohormone]_xylem_) and its delivery to the shoot (Phytohormone_delivery_) measured in either part of the root zone (wet side and dry side). ABA = Abscísic acid. JA= Jasmonic acid. ACC= 1-Aminocyclopropane-1-carboxylic acid. Plants girdled in the wet side of the plant were excluded in *ANOVA 1*, while those girdled in the dry side were excluded from *ANOVA* 2. In bold, significant *P*-values (*P*<0.05)

|  | **[ABA]_xylem_** | |  | **[JA]_xylem_** | | |  | **[ACC]_xylem_** | |  | **ABA_delivery_** | |  | | **JA_delivery_** | | | **ACC_delivery_** | | |  |
| --- | --- | --- | --- | --- | --- | --- | --- | --- | --- | --- | --- | --- | --- | --- | --- | --- | --- | --- | --- | --- | --- |
|  | *P-value ANOVA1* | *P-Value ANOVA2* |  | *P-value ANOVA1* | *P-Value ANOVA2* |  | | *P-value ANOVA1* | *P-Value ANOVA2* |  | *P-value ANOVA1* | *P-Value ANOVA2* | |  | | *P-value ANOVA1* | *P-Value ANOVA2* |  | *P-value ANOVA1* | *P-Value ANOVA2* | |
| *Wet side* |  |  |  |  |  |  | |  |  |  |  |  | |  | |  |  |  |  |  | |
| Irrigation (I) | 0.37 | 0.48 |  | **0.008** | **0.009** |  | | 0.37 | 0.48 |  | 0.20 | 0.22 | |  | | **0.002** | **0.003** |  | **<0.001** | **0.007** | |
| Girdling (G) | 0.28 | 0.29 |  | 0.66 | 0.81 |  | | 0.28 | 0.29 |  | 0.24 | 0.26 | |  | | 0.50 | 0.62 |  | 0.21 | 0.06 | |
| Day (D) | 0.76 | 0.71 |  | 0.49 | 0.55 |  | | 0.76 | 0.71 |  | 0.70 | 0.67 | |  | | 0.15 | 0.13 |  | 0.26 | 0.11 | |
| I x G | 0.68 | 0.53 |  | 0.99 | 0.81 |  | | 0.68 | 0.53 |  | 0.44 | 0.47 | |  | | 0.68 | 0.60 |  | 0.11 | **0.04** | |
| I x D | 0.50 | 0.44 |  | 0.34 | 0.15 |  | | 0.50 | 0.44 |  | 0.59 | 0.55 | |  | | 0.09 | 0.10 |  | 0.49 | 0.39 | |
| G x D | 0.49 | 0.41 |  | 0.73 | 0.90 |  | | 0.49 | 0.41 |  | 0.40 | 0.44 | |  | | 0.93 | 0.82 |  | 0.83 | 0.74 | |
| I x G x D | 0.90 | 0.87 |  | 0.81 | 0.77 |  | | 0.90 | 0.87 |  | 0.82 | 0.77 | |  | | 0.80 | 0.75 |  | 0.80 | 0.62 | |
| *Dry side* |  |  |  |  |  |  | |  |  |  |  |  | |  | |  |  |  |  |  | |
| Irrigation (I) | **<0.001** | **<0.001** |  | 0.72 | 0.84 |  | | **<0.001** | **<0.001** |  | **<0.001** | **<0.001** | |  | | 0.77 | 0.85 |  | **0.01** | **0.04** | |
| Girdling (G) | 0.30 | 0.48 |  | 0.97 | 0.93 |  | | 0.30 | 0.48 |  | 0.15 | 0.44 | |  | | 0.98 | 0.99 |  | 0.41 | 0.79 | |
| Day (D) | 0.87 | 0.97 |  | 0.42 | 0.74 |  | | 0.87 | 0.97 |  | **0.04** | 0.22 | |  | | 0.20 | 0.14 |  | **0.02** | 0.06 | |
| I x G | 0.23 | 0.33 |  | 0.78 | 0.88 |  | | 0.23 | 0.33 |  | 0.13 | 0.41 | |  | | 0.79 | 0.68 |  | 0.93 | 0.56 | |
| I x D | 0.79 | 0.99 |  | 0.98 | 0.70 |  | | 0.79 | 0.99 |  | 0.05 | 0.27 | |  | | 0.47 | 0.72 |  | 0.11 | 0.17 | |
| G x D | 0.68 | 0.56 |  | 0.74 | 0.73 |  | | 0.68 | 0.56 |  | 0.10 | 0.37 | |  | | 0.69 | 0.99 |  | 0.34 | 0.53 | |
| I x G x D | 0.66 | 0.49 |  | 0.97 | 0.50 |  | | 0.66 | 0.49 |  | 0.11 | 0.37 | |  | | 0.71 | 0.81 |  | 0.77 | 0.64 | |

**Supplementary Table 4.** Results of the analyses of variance (*P*-values) for total phytohormone delivery to the shoot (Phytohormone_delivery_). ABA = Abscísic acid. JA= Jasmonic acid. ACC= 1-Aminocyclopropane-1-carboxylic acid. Plants girdled in the wet side of the plant were excluded in *ANOVA 1*, while those girdled in the dry side were excluded from *ANOVA* 2. In bold, significant *P*-values (*P*<0.05)

|  | **ABA_delivery_** | | | |  | **JA_delivery_** | | |  |  | **ACC_delivery_** | | |  | |  | | |  |
| --- | --- | --- | --- | --- | --- | --- | --- | --- | --- | --- | --- | --- | --- | --- | --- | --- | --- | --- | --- |
|  | *P-value ANOVA1* | | *P-Value ANOVA2* | |  | *P-value ANOVA1* | | *P-Value ANOVA2* | | *P-value ANOVA1* | | | *P-Value ANOVA2* | |  | |  | | |
| Irrigation (I) | | 0.69 | | 0.87 |  | **<0.001** | **<0.001** | |  | 0.08 | | 0.06 | | | | | |  |  |
| Girdling (G) | | **0.008** | | **0.008** |  | 0.08 | 0.06 | |  | 0.81 | | 0.78 | | | | | |  |  |
| Day (D) | | **<0.001** | | **<0.001** |  | **0.05** | 0.05 | |  | 0.10 | | 0.15 | | | | | |  |  |
| I x G | | 0.74 | | 0.48 |  | 0.07 | 0.06 | |  | 0.99 | | 0.99 | | | | | |  |  |
| I x D | | 0.96 | | 0.80 |  | **0.05** | 0.06 | |  | 0.18 | | 0.36 | | | | | |  |  |
| G x D | | 0.25 | | 0.43 |  | 0.21 | 0.13 | |  | 0.91 | | 0.99 | | | | | |  |  |
| I x G x D | | 0.70 | | 0.78 |  | 0.25 | 0.19 | |  | 0.77 | | 0.96 | | |  |  |  |  |  |
